# Constructing the Quality Measurement Model of Street Space and Its Application in the Old Town in Wuhan

**DOI:** 10.3389/fpubh.2022.816317

**Published:** 2022-02-24

**Authors:** Tianyue Wan, Wei Lu, Peijin Sun

**Affiliations:** Research Section of Environment Design, School of Architecture and Fine Art, Dalian University of Technology, Dalian, China

**Keywords:** street space, physical environment, pedestrian perception, comprehensive measurement, validity test

## Abstract

The quality of street space is the comprehensive suitability evaluation from the objective physical environments and the subjective pedestrian perception. Since the existing quality measurement models of street space do not consider both subjective and objective aspects, it is difficult for planners to accurately locate the low-quality streets that need to be regenerated. To solve this problem, this study proposes a new 5D+3S measurement model for street space quality evaluation. This model incorporates the widely acknowledged 5D dimensions of the physical environments (Design, Destination accessibility, Distance to transit, Density, and Diversity), and the 3S dimensions (Sociality, Safety, and Status) of walking perception derived from the keywords clustering on relevant literature. To test the validity of the proposed model, this study makes a comparative analysis on the results of the public assessment, expert scoring, and model measurement to verify whether the measurement results are objective and convincing. The results show that the quality grade obtained by the proposed measurement model is highly consistent with the subjective evaluation outcomes of the public and experts. Thus, the proposed measurement model is effective in quality measurement of the street space, which provides a new idea for future large-scale diagnosis of city public space quality.

## Introduction

The street is a pluralistic public space that integrates society, economy, and culture. It is the result of an institutionalized human movement and has the economic and social significance that extends beyond the entity identity (1). Their spatial quality reflects the user's assessment of the space's superiority or inferiority, which is influenced by personal values and spatial perception (2). Street space has evolved from a linear three-dimensional space between buildings (3) to a place where space emphasizes urban humanistic vitality and natural factors (4–6). Many cities and institutions propose street scores, walkability indexes, and walk scores for a high-quality street space. These measurements help residents and designers to accurately judge the quality of the street space and to serve as a guide for later decisions about how to improve it.

Sustainable development goals (SDGs) 11.7 proposes opportunities to provide universal access to safe, inclusive, accessible, green, and public space, particularly for women, children, the elderly, and disabled people (7). According to UN-report Habitat's STREETS AS PUBLIC SPACE AND DRIVER OF URBAN PROSPERITY, the majority of public spaces in cities are street spaces (8). Street design guidelines have been issued in cities like Copenhagen and London. However, most cities lack clear standards to measure the quality of a street space. At the moment, research on the quantitative measurement of street space is primarily focused on urban morphology, environmental behavior, and data science, *via* field research, significant data acquisition, or machine learning methods to obtain and to analyze data (9–11). However, recent research on the quality of urban street space emphasizes the availability of data and technology. The measurement of street space perception, from the perspectives of the users, meets efficiency and refinement requirements, but it lacks comprehensive quality measurement systems. The measurement of street space quality using an objective physical environment and a non-objective subjective pedestrian perception is still a challenge. Additionally, current research on the quality of street space measurement focuses exclusively on the index system's construction, data collection, and processing, omitting verification of the measurement results' credibility or measurement model's validity. However, rather than a standardized measure of tangible space, spatial quality is a value judgment combining subjective and objective values under existing norms. As a result, given the abstract and invisible nature of street space, using reliable standards for result verification remains a challenge for planners and designers.

Big data and artificial intelligence are now widely used in urban spatial research, overcoming traditional spatial data acquisition and analysis limitations. It helps build a more comprehensive street space quality measurement model.

This study aims to develop a comprehensive quality measurement model of street space by integrating multiple influencing factors; the model is then applied to streets in the old town and the model's effectiveness is verified from objective and subjective levels. The main issues are as follows: (1) How to construct a measurement model for evaluating the quality of street space including the objective physical environments and subjective pedestrian perception? How to select the appropriate measurement factors? (2) How to verify the effectiveness combined with the subjective evaluation of people? Are the measurement results of the model consistent with the subjective evaluation results?

This study combs the concept, system, and technical quality measurement methods about street space in Section Literature Review. The methods for constructing the 5D+3S quality measurement model of street space and model validation are introduced in Section Literature Review. Then, Section Literature Review summarizes and analyzes the relevant measurement and verification results. The last section summarizes this study and demonstrates the applicability and limitations of the current study.

## Literature Review

### Defining the Measurement of Street Space Quality

Streets are spatial entities that are associated with vehicular traffic and material flow, and have a diverse and complex social context (12). A quality is the attribute of an object or its property characteristics as assessed by intended users. So, the quality of street space reflects the subjective evaluation based on the principle that users meet their quality requirement for life, production, and development (13, 14). Different from a mechanized urban space design, Jacobs (15), Gehl (16), and Whyte (17) et al. emphasized the relationships between social activities and spatial perceptions from the perspective of public life. The relevant literature focuses on two aspects of measuring the quality of street space: physical environment and pedestrian perception (9, 18, 19).

In the measurement of the physical environment, academic works have proven that street scale (20), connectivity (21), building density (22), traffic convenience (23), and diversity of public service facilities (24) are the primary factors affecting the quality of a physical environment. For example, the UN-Habitat global public space assessment toolkit measures the quality of the built environment of the street from 12 indicators, such as street intersection, pedestrian path, street greening, and motor vehicle lane (25).

In the measurement of pedestrian perception, a considerable number of studies have measured the perceived qualities of street space, including street safety (26), visual landscape (27), space attraction (28), and activities in street space (9). For example, Carmona measured the perspective of pedestrians from accessibility, attractiveness, comfort, inclusiveness, vitality, uniqueness, and safety (6). However, there is a scarcity of research on the quality measurement of street space that incorporates the two aspects mentioned above.

Nowadays, China's urban construction focuses on urban micro renewal and humanistic design. The function of street space has changed from car centered to people-oriented. Meanwhile, new big data types and machine learning algorithms are widely used in street space research extending the original physical space measurement dimension. Based on the dual connotation of street space quality, the new requirements for urban renewal, and the widespread use of new data processing technology, this study integrates the objective physical environment and the subjective pedestrian perception space for a comprehensive quality measurement.

### Major Studies on Measuring Street Space Quality

#### Measurement Systems of Street Space Quality

Based on defining the measurement of street space quality, the measurement systems have mainly revolved around two aspects: physical environment and pedestrian perception. These are generally addressed in Table 1.

**Table 1:** Representative studies for the quality measurement system of street space.

**Dimension**	**Category**	**Case study**	**Measurement system**
A. Physical environment	(1) Functionality	Cervero and Kockelman (29)	The 3D system: design, density, and diversity.
	(1) Functionality (2) Serviceability	Active Living Research (23)	Microscale audit of pedestrian Streetscapes: crossing facility convenience, street space design, space art aesthetics, walking path quality, and bus facility stop.
		Moura et al. (30)	The 7C system: facility coexistence, spatial comfort, daily management, street connectivity, landmark buildings or facilities, spatial convenience, and social.
	(1) Functionality (2) Architecture	Oliveira (31)	Spatial morphology measurement system: block scale, building function, building age, building alignment rate, street accessibility, and plot density.
	(1) Functionality (2) Architecture (3) Demography	Sevtsuk and Mekonnen (32)	Urban spatial network measurement system: population density, building function, building strength, and building quality, street accessibility.
	(1) Functionality (2) Diversity (3) Demography (4) Architecture	Ewing and Cervero (24)	The 5D system: design, density, diversity, distance to transit, and Destination accessibility.
B. Pedestrian perception	(1) Psychology	Ewing and Handy (33)	Measuring psychology of pedestrian from image ability, enclosure, human scale, transparency, and complexity.
	(1) Vision	Dubey et al. (34)	Measuring perceptual attributes of pedestrian activity: safe, lively, dull, rich, depressed, and beautiful.
	(1) Vision (2) Auditory	Li et al. (35)	Measuring pedestrian comfort through visual elements and corresponding auditory elements, including water flow, plants, birds, human activities.
	(1) Vision (2) Psychology	Porta and Renne (28)	Measuring four subjective indicators: pedestrian walking emotion, sense of enclosure, security, social interaction, and attractiveness.
	(1) Vision (2) Auditory (3) Touch (4) Olfaction	Rezvanipour et al. (36)	Measuring pedestrian perception from five aspects: street comfort, pleasant walking pleasure, physical safety, environmental safety, and criminal safety.

In terms of measuring the physical environment of streets, the measurement systems involve one or more of the five categories of street functionality, diversity, serviceability, demography, and architecture (37–39), which have been widely confirmed in academic discussions. However, the applicability of different measurement systems in specific empirical studies remains to be discussed. To effectively assess the impact of physical environmental quality on residents' lives, measurement factors must be selected based on the area's actual situation.

In terms of measuring the pedestrian perception of streets, academic discussions and applications have applied the comprehensive multisensory measurement from sensory stimulation and environment cognition, and the dimensions of measuring perspectives are divided into five categories: psychology, vision, auditory, touch, and olfaction (28, 33–36, 40). However, pedestrian activity behavior may change in a controlled environment, and the results may not accurately represent the sensory feedback in the actual streets (41, 42).

#### Measurement Technologies of Street Space Quality

The measurement technologies of physical environment are based on spatial data that include a traditional three-dimension spatial data, location-based services data (LBS), point of interest (POI) data, and transportation data. Yang et al. (43) used LBS and POI data to calculate the hill number in ecology for determining the degree of spatial mixing. Zhang et al. (44) used a multi-source urban geospatial big data to analyze Baidu's POI data and real-time Tencent user density for the degree of crowding. But, these measurement technologies still face some problems, such as difficult data acquisition, high cost, insufficient accuracy, operability of measurement technology, and so on.

The pedestrian perception measurement technologies are classified into three categories: traditional questionnaire survey, emotional analysis of social media data, and physiologic perceptron analysis. Rollero and Piccoli (45) used traditional questionnaires to investigate the environmental perceptions of citizens. Alexander et al. (46) used a random sample of Swedish adults to test 17 issues that were related to local street aesthetics, safety, and society. The following paragraphs discuss the last measurement technologies, respectively.

First, in terms of social media data analysis technologies, some researchers use semantic analysis to analyze the emotional characteristics of pedestrians in social media data such as text classification, sentiment analysis, and intention recognition. For example, Kovacs-Györi et al. (47) used Twitter data to extract the emotions of pedestrians, and analyze the characteristics of emotions over time. Plunz et al. (48) used a Twitter database with geolocation information to analyze the emotional impact of green spaces on pedestrians.

Second, physiological perception analysis is widely using street view images to measure the pedestrian visual perception, and uses a fully convolutional neural network to segment the street view images (49). Several studies utilized biosensors to evaluate the pedestrian response in different street spaces, including Electroencephalogram (EEG), Eye-tracking Metric (ETM), and pressure tests. These technologies provide new technical references for measuring street space quality. For example, Kim et al. (50) used EEG to assess the relationship between the walking environment and pedestrian perception at night.

Therefore, the measurement technologies of street space quality are based on qualitative analysis and quantitative expression. Machine learning technologies are used to analyze the correlation between street space quality and natural environment, social economy, pedestrian physiological characteristics, and other factors. However, whether the current pedestrian perception measurement methods are equivalent to the actual pedestrian perception of walking is still worth discussing.

### Validity Test of Measurement Models

Verifying the relationship between measurement results and human subjective evaluation is still a difficulty in the study field. A few researchers have studied the validity of the measurement model from two aspects: subjective verification, subjective and objective comparison verification.

#### Subjective Verification

Academic studies mainly adopt the Experts Grading Method to verify the measurement results. Although the rating scale and classification method have subjective deviations, the result is generally considered acceptable, and is highly effective in certain situations (51). For example, Rezvanipour et al. (36) invited experts in different fields to evaluate the relationship between the physical environment and social attributes of the street from the aspects of urban policy, social behavior, and urban aesthetics. Ewing and Handy et al. (33) established an expert evaluation team to qualitatively define the quality of street space and to score different street scenes. While the results of expert evaluation can represent the standardization of space design, they may not accurately reflect the perspectives of participants in urban sociology and environmental behavior research.

#### Subjective and Objective Comparative Verification

Subjective and objective comparative verification methods are mainly divided into two types: public evaluation and machine learning comparative verification, and expert evaluation and machine learning comparative verification. Both methods use deep learning algorithms such as convolutional neural networks to train sample data sets, and to analyze the consistency between the classification results of the samples and the subjective evaluation results. For example, Liu et al. (52) used the method of expert rating to label images and train the models, then, conducted a field survey on pedestrians and compared the public's rating scores with the machine rating scores. Ye et al. (53) used SegNet to extract the design elements that affect the quality of street space from street view images, produced a Java-based program to collect expert preferences through pairwise comparison, and evaluated the effectiveness of the model through ANN training.

Based on the above discussion, there is a lack of comprehensive subjective evaluation of street space quality from the two levels of expert scoring and public evaluation. In addition, whether the results obtained by training the model only with street view images can reflect the overall quality of street space needs to be verified. Therefore, this study avoids the defects of current effectiveness testing, and comprehensively verifies the effectiveness of the measurement results through six aspects: public evaluation, street classification, expert scoring, data collection, model measurement, and result comparison.

According to the previous studies, this research constructs a multi-dimensional systematic quality measurement model of street space, and comprehensively verifies the effectiveness of the measurement results from subjective and objective.

## Methodology

### The Theoretical Framework

**Figure 1:**
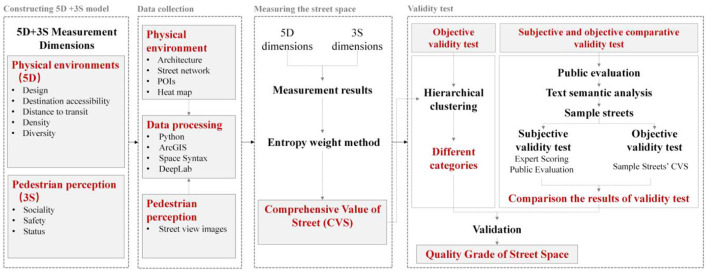
The theoretical framework.

The theoretical framework points to four stages in this study: constructing a measurement model, data collection, measuring the quality of street space, and validity test (Figure 1). The research questions tackled in this study link the objective physical environment with the subjective perception of an individual. All the main issues in this study are related to the 5D+3S measurement model.

### Study Area and Data Collection

#### Study Area

The old town of Wuchang is located in the central of Wuhan City, with a total area of 7.7 square kilometers (Supplementary Figure 1). It had maintained the first-class administrative center for more than 600 years from the Yuan Dynasty to the Qing Dynasty. The old town has a clear road network (Supplementary Figure 2), but the north-south dual-city pattern is naturally formed due to the obstruction of Snake Mountain. Some traditional street textures are retained in the north, and new urban roads are mainly in the south.

#### Physical Environment Data

This study applies Baidu Maps API to collect the architecture data, street network data, POI data, and heat map data. The ArcGIS analysis platform processes the original architecture data and simplifies the street networks (Supplementary Figure 3). The 62 streets and 215 street sections were determined as research objects. Concerning the classification of national economic industries, regional functions, and the development of the old town, this study selects 3,066 POI data including five categories: commercial services, public management and public services, transportation facilities, residential buildings, and green spaces. This study collects the 112 rasterized heat maps of each hour from 7:00 to 23:00, between June 1 and 7 in 2021. And, by calculating the average heating value at different time points in each street section, the clustering of active people in the street space is evaluated.

#### Pedestrian Perception Data

According to the Detailed Regulations for Wuhan Urban Design Management, the neighborhood units in the old town are divided into three levels. The sampling points for collecting street view images are selected according to the different levels of the neighborhood (Supplementary Table 1).

**Figure 2:**
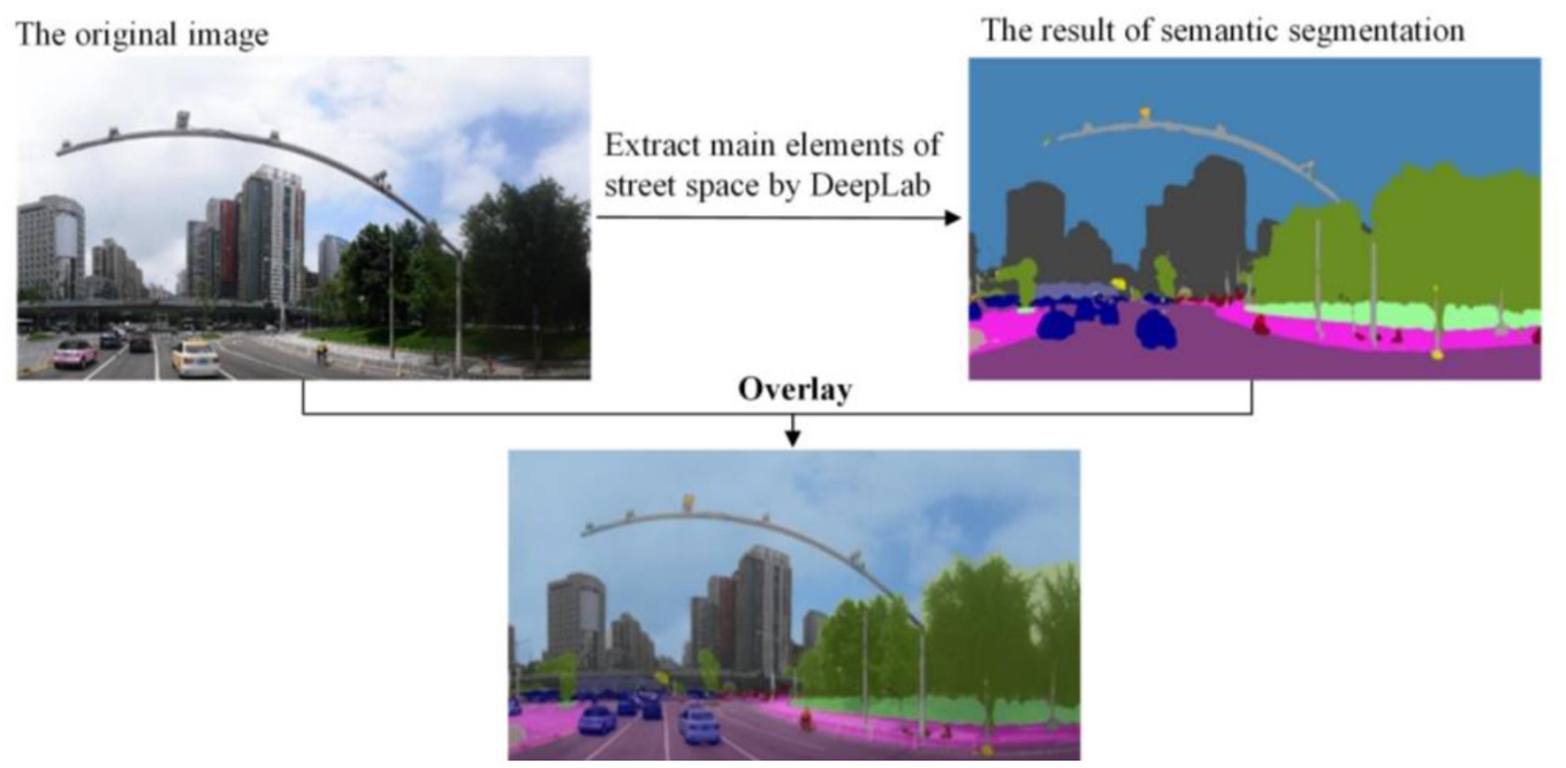
Schematic diagram of street view image processing.

This study uses the DeepLab V3 model (54) trained on the Cityscapes dataset (55) to semantically segment the panorama image for obtaining the proportion of street elements. Limited by the computing device and the size of the collected panorama image dataset, the pre-trained DeepLab model is finetuned on our street view dataset using a transfer learning strategy and achieves mIoU of about 78.8%. The finetuning process is implemented on the open-source deep learning framework TensorFlow (56). Some studies (57, 58) have also illustrated that this segmentation model performed well in Chinese cities. It can segment the panorama into 20 categories, such as sky, road, vegetation, and vehicles, to achieve a pixel-level image segmentation and extract various street space elements (Figure 2). Through the statistical analysis of the segmentation results, the proportion of research elements is obtained, so the segmentation results corresponding to each street scene sampling point are connected with the street section.

### Selection of Specific Measurement Indicators

#### 5D+3S Measurement Model Basic Framework

When constructing the measurement model, this study mainly expounds from three aspects: index selection, selection reasons, and calculation methods. First, the measurement dimensions include the physical environment and pedestrian perception according to the concept of street space, while the specific measurement levels, indicators, and factors are determined by nature, culture, architecture, economy, infrastructure, and residents. Then, when considering the specific measurement level, a secondary selection of measurement indicators is carried out based on the world's established indicators and literature studies. Finally, the selected measurement indicators are subdivided to define the measurement factors and quantitative methods.

At the dimension of physical environment, many quality measurement systems of street space are aimed at the material space elements. Given the comprehensiveness of the measurement and the comparison of other measurement index systems, the 5D index proposed by Ewing and Cervero has been widely confirmed in the built environment measurement research (29). So, this study focuses on the Design, Density, Diversity, Destination accessibility, and Distance to transit in the 5D index as the physical environment dimension measurement levels in the quality measurement model (24).

From the pedestrian perception dimension, this study clustering literature about street space vitality and pedestrian perception from 2001 to 2021 by CiteSpace software show results that research on the subjective perception of street space mainly focuses on streets' sociality, safety, and status (Supplementary Figure 4).

Based on the above analysis, this study determined the measurement levels in the physical environment dimensions are 5D (Design, Destination accessibility, Distance to transit, Density, and Diversity). Then, the 3S pedestrian perception levels are Sociality, Safety, and Status. In the 5D+3S measurement model, a series of representative measurement index lists are selected to comprehensively evaluate the spatial quality of streets (Table 2).

**Table 2:** The 5D+3S measurement model framework.

**Dimension**	**Measure level**	**Measure index**	**Measure factor**	**Calculation formula**
5D	Design	Natural environment	Green view index (GVI) (7, 59, 60)	$GVI_{n}=\frac{\sum_{i=1}^{i}g_{i}}{\sum_{i=1}^{i}a_{i}}$
			Sky openness (SO) (61)	$SO_{n}=\frac{\sum_{i=1}^{i}s_{i}}{\sum_{i=1}^{i}a_{i}}$
		Build environment	Street integration (SI) (62)	$SI_{n}=\frac{1}{R_{n}}=\frac{m\left[\log_{2}\left(\frac{m+2}{3}-1\right)+1\right]}{\left(m-1\right)|\bar{D}-1|}$
			Street connectivity (SC) (63)	*SC*_*n*_ = *k*_*n*_
	Destination accessibility	Traffic accessibility	Traffic accessibility (TA) (64, 65)	$TA_{n}=\frac{1}{n}\overset{n}{\underset{i=1}{\sum }}\left(\frac{1}{n-1}\overset{n}{\underset{\begin{array}{c} j=1\\ j\neq i \end{array}}{\sum }}\left(d_{ij}\right)\right)$
			Network accessibility (NA) (66)	$NA_{k}=\frac{\sum_{i=1}^{i}l_{i}}{\sum_{i=1}^{i}s_{i}}$
		Accessibility of public facilities	Facilities accessibility (FA) (43)	*FA*_*i*_ = *N*_*i*_
	Distance to transit	Distance from underground traffic	Distance to subway (DS) (67, 68)	$DS=Nearest\text{\_}Dis\left(\sqrt{{\left(x_{r}-x_{s}\right)}^{2}+{\left(y_{r}-y_{s}\right)}^{2}}\right)$
		Distance from ground traffic	Distance to bus-station (DB) (67, 68)	$DB=Nearest\text{\_}Dis\left(\sqrt{{\left(x_{r}-x_{b}\right)}^{2}+{\left(y_{r}-y_{b}\right)}^{2}}\right)$
	Density	Population density (4, 69)	Heatmap on weekday (HW)	$HW={\bar{H}}_{1}=\frac{\sum H_{i}}{m}$
			Heatmap on rest-day (HR)	$HR={\bar{H}}_{2}=\frac{\sum H_{i}}{m}$
		Building density	Floor-area Ratio (FR) (20)	$FR=\frac{S_{a}}{S_{l}}$
	Diversity	Diversity of public facilities	Number of public facilities (NPF) (29, 43, 69)	$NPF=\sum_{i=1}^{i}p_{i}$
			Mixture of public facilities (MPF) (70, 71)	$LCA_{n}=\frac{\sum_{i=1}^{i}\left(p_{i}+r_{i}\right)}{\sum_{i=1}^{i}a_{i}}$
3S	Sociality	Social crowd (72, 73)	Low-speed crowd agglomeration (LCA) (74)	$MPF=-\overset{n}{\underset{ij=1}{\sum }}p_{ij}Inp_{ij}$
	Safety	Individual safety	Individual safety (IS) (4, 75, 76)	$TS_{n}=\frac{\sum_{i=1}^{i}m_{i}}{\sum_{i=1}^{i}a_{i}}$
		Traffic safety	Traffic safety (TS) (51, 77)	$HCL_{n}=\frac{\sum_{i=1}^{i}S_{h}}{\sum_{i=1}^{i}S_{a}}$
	Status	Regional culture (78)	Historical and cultural land (HCL)	$IS_{n}=\frac{\sum_{i=1}^{i}p_{i}}{\sum_{i=1}^{i}a_{i}}$
			Cultural atmosphere (CA)	$CA_{n}=Nearest\text{\_}Dis\left(\sqrt{{\left(x_{r}-x_{c}\right)}^{2}+{\left(y_{r}-y_{c}\right)}^{2}}\right)$
		Regional landscape	Landscape visibility (LV) (79, 80)	$LV_{n}=\frac{S_{v}\cap S_{l}}{S_{v}}$

#### Calculation of Comprehensive Quality Measurement Value of Street Space

There are 20 measurement factors in this study, and the properties and dimensions between the factors are different. When the values of the index measurement differ significantly, the impact of the high-value index on the comprehensive analysis increases, resulting in a deviation of the results. Therefore, this study selects the min-max data standardization method to re-scale the raw data without changing the original data structure.

To avoid the inaccuracy of the index assignment results caused by subjective evaluation method factors such as personal emotion and cognitive bias (70, 71), this study uses the entropy weight method to objectively assign the index weight. The entropy weight method (EWM) is a well-known and widely used information weight model. In comparison to other subjective weight models, the primary advantage of EWM is that it eliminates human factor interference with index weights, thereby increasing the objectivity of the results of the comprehensive evaluation (81). The degree of differentiation is used to determine the value in EWM. The greater the dispersion of the measured value, the greater the differentiation of the indicator, and thus, the more information that can be derived.

Then, the entropy weight method is applied to calculate the weight (λ_*i*_) of each index to calculate the Comprehensive Value of Street (CVS). According to the weight of each measurement index, the comprehensive quality λ_*i*_ measurement value formula of street space is as follows:

$$\begin{array}{l} CVS=0.254\times GVI+0.252\times SO+0.104\times SI+0.378\times SC\\ \;+0.055\times TA+0.035\times CA+0.114\times FA\;+0.095\times DS\\ \;+0.052\times DB+0.033\times HW+0.036\times HR+0.044\times FR\\ \;+0.401\times NPF+0.486\times MPF\;+0.170\times PC+0.073\\ \;\times +IS0.004\times TS+0.382\times HCL+0.238\times CA+0.133\\ \;\times LA \end{array}$$

### Validity Test of 5D+3S Measurement Model

#### Objective Validity Test

To verify the effectiveness of the proposed 5D+3S quality measurement model of street space, a hierarchical clustering algorithm is applied to automatically analyze each street index data without human intervention by automatically dividing the street data with similar spatial quality into a cluster. This clustering algorithm is conducted with open-source Python libraries, SciPy, and Scikit-learn. In the initial stage, each record in the street space dataset is regarded as a separate cluster. Then, these small clusters are hierarchically merged into bigger clusters according to the distance criterion with the iteration of the clustering algorithm. In this study, considering the comparison results of some common similarity measurement methods, such as single, average, Ward, and so on, we found that the clustering results obtained by the Ward method are the most content with our data characteristics. In addition, some existing studies (82, 83) also prove that the Ward method had a better performance and robustness. Thus, the Ward minimization variance method is adopted to implement the clustering algorithm, which is formulated as follows:

$$\begin{array}{l} d\left(u,v\right)=\sqrt{\frac{\left|v|+|s\right|}{T}d{\left(u,s\right)}^{2}+\frac{\left|v|+|t\right|}{T}d{\left(u,t\right)}^{2}-\frac{\left|v\right|}{T}d{\left(s,t\right)}^{2}} \end{array}$$

where *s* and *t* are clusters, *u* is a new cluster composed of *s* and *t*, and *v* is an unused cluster, *T* = |*v*| + |*s*| + |*t*|, and |*| represents the number of data records in the cluster.

According to the concept of hierarchical clustering algorithm, the hierarchical clustering algorithm does not require a predefined number of clusters, and the number of clusters does not affect the final result of the validity test. So, this study clusters the CVS into three categories (good, medium, and poor) as a simple example of 5D+3S measure model validity test. All the data records are assigned with corresponding category labels, taking the three largest clusters as the final clustering results. Then, further analysis of the original results of the proposed 5D+3S measurement model is implemented through other visualization methods. Furthermore, the clustering results and the comprehensive values of the proposed space quality measurement model are also compared to analyze whether the measurement index values in the proposed model are effective.

#### Subjective and Objective Comparative Validity Test

Using the selected sample streets as the test objects, this study uses an expert scoring and public sentiment polarity in the social media big data to determine the subjective evaluation results. The CVS is taken as the result of objective verification, which is calculated using the 5D+3S quality measurement model of the street space. By comparing the two verification results, to determine whether the measurement model is consistent with subjective evaluations of people and whether it is capable of reflecting the problems with the street space quality from the perspective of the public.

**Figure 3:**
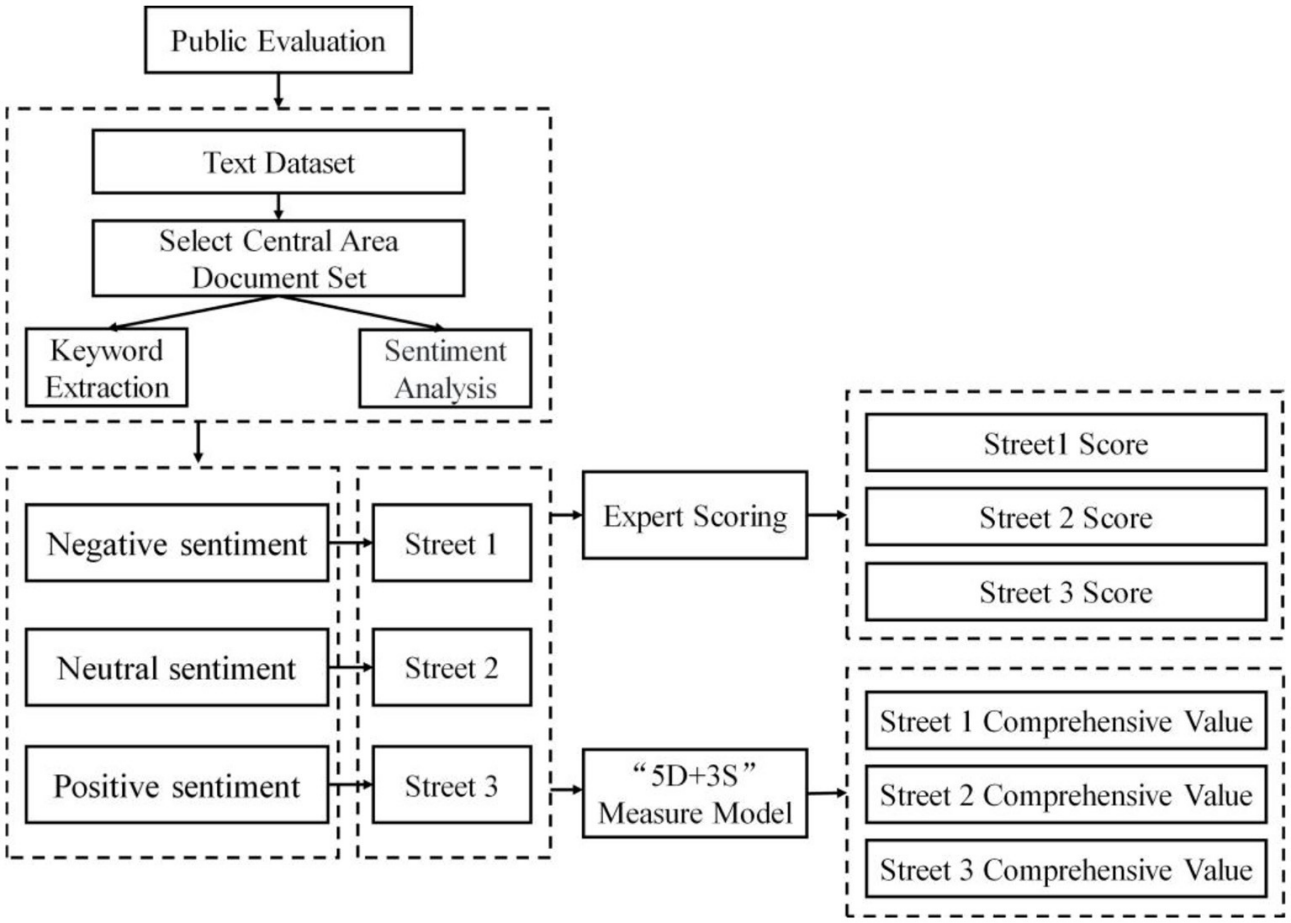
Technical framework diagram of validity detection.

To avoid the cognition bias caused by the randomly selected residents to score the streets, this study collects the Weibo check-in data, public comment data, and Zhihu Q&A data from June 2020 to December 2020 in the central area of Wuhan, and applies a sentiment analysis to evaluate the quality of different street space from public perception. The public evaluation data are processed by two steps: the high-frequency words extraction according to TF-IDF (84) and the sentiment analysis (85, 86) based on the high-frequency words. Then, the three streets with different sentimental evaluations are selected as sample streets for expert scoring, and the scoring criteria are supported by Street Planning and Design Guidelines in Wuhan. In addition, this study collects the data of physical environment and pedestrian perception about sample streets for objective measurement in 5D+3S model. Finally, the result of 5D+3S measure model, public evaluation, and expert scoring are compared to analyze the relationship between these three results. The specific technical framework is shown in Figure 3.

## Results

### Comprehensive Quality Measurement Results of Street Space

**Figure 4:**
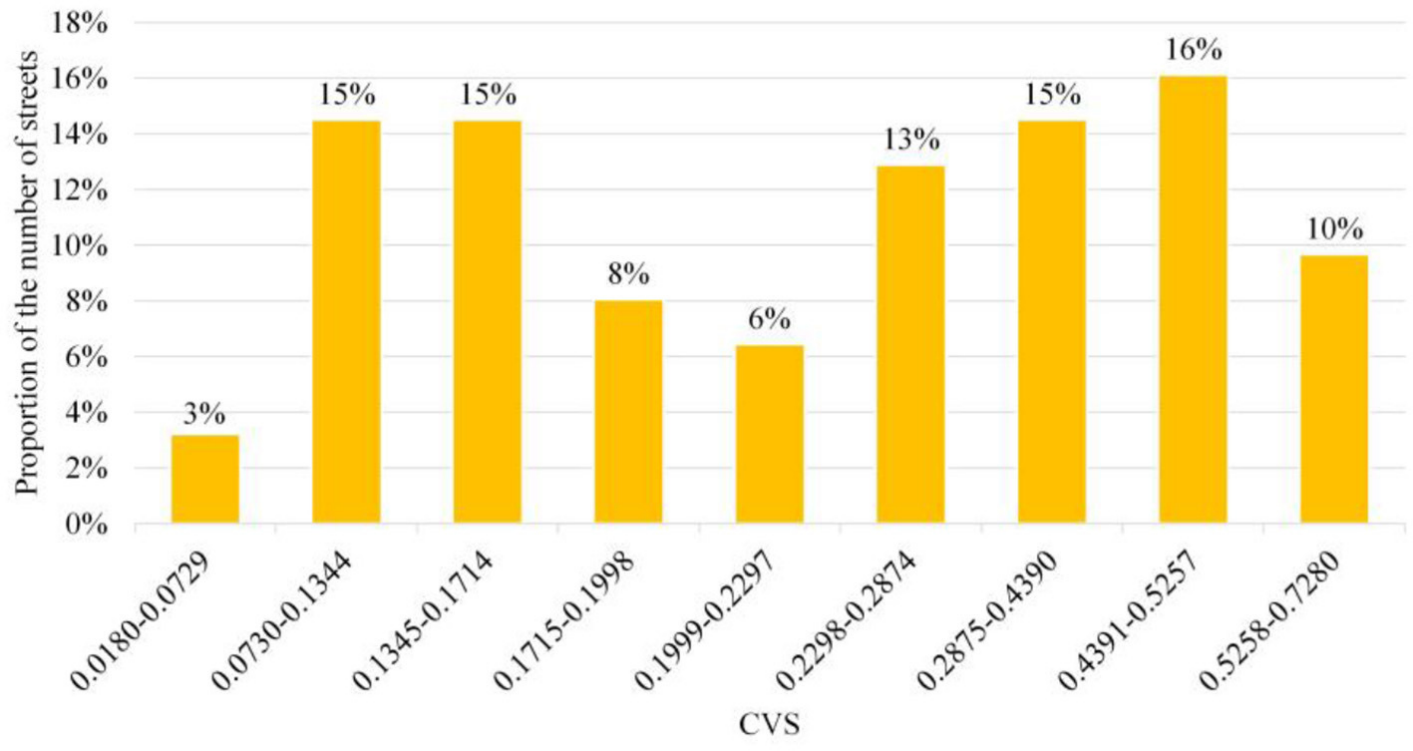
The street proportion of CVS within the interval of Jenks natural breaks classification.

In order to research the situation of quality distribution, this study takes the street space as the basic unit to obtain the CVS through a numerical standardization and weighted assignment of measurement indicators at all levels. From the perspective of the overall value, the CVS in Wuchang is distributed within the range of [0.018, 0.728], and the mean is 0.251. By calculating the Goodness of Variance Fit (87, 88) (GVF) of the CVS, it can be known that when the number of classifications is equal to 9, it is the inflection point of the GVF curve (89). Therefore, this study uses the natural breaks method in ArcGIS to divide the CVS into 9 categories. The classification results and the proportion of streets in different numerical intervals are shown in Figure 4. It can be seen that the number of streets with low and high comprehensive measure values is quite more, while the number of streets with medium values is less. Thus, the CVS of different streets in the old town is considerably different, showing a polarization trend.

**Figure 5:**
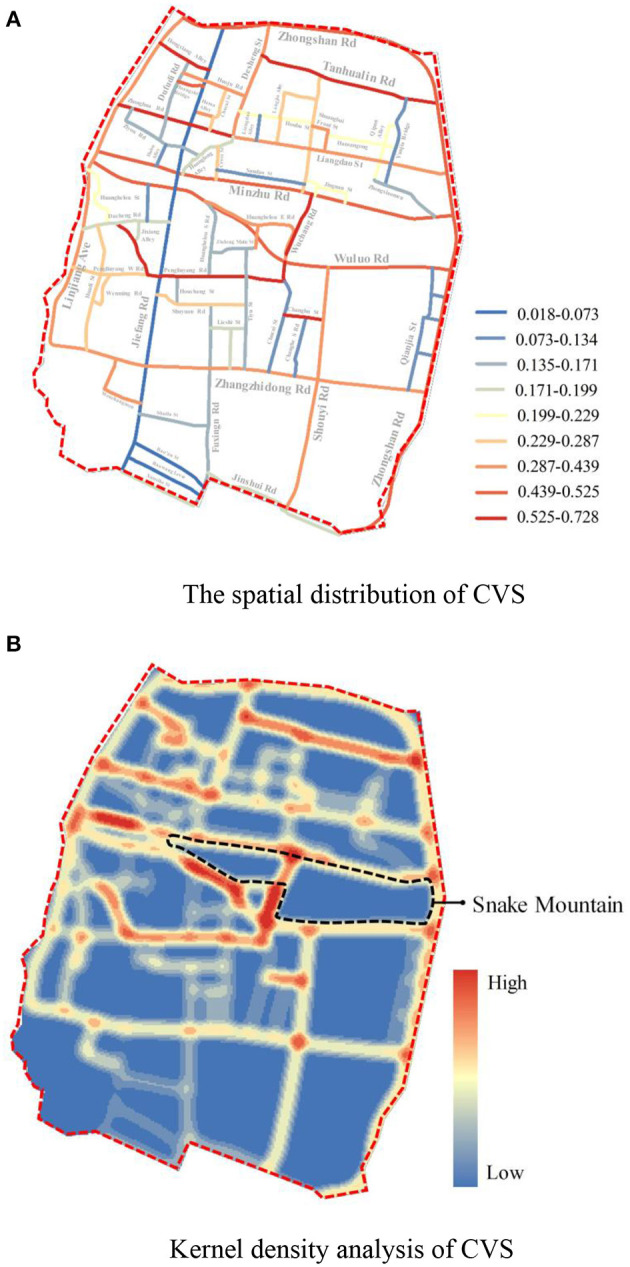
The spatial distribution and kernel density analysis of comprehensive value of street (CVS). **(A)** The spatial distribution of CVS. **(B)** Kernel density analysis of CVS.

This study analyzes the kernel density of the CVS to analyze the spatial distribution characteristics of different street space qualities in the old town. It divides the grid data of the results of nuclear density analysis into seven levels for visual expression. In Figure 5, it can be found that the north of the Snake Mountain has more streets with a higher CVS and densely distributed high-value points than the south and the other regions, which indicates that high-quality street spaces gather in the area north of the Snake Mountain.

According to the analysis of the overall pattern of street space quality distribution, the low-value CVS points are concentrated in the area south of Snake Mountain. This area is still under construction, and most roads are new. Streets have lost their humanistic vitality due to the standardization and massive construction. Concurrently, the construction site, road closures, and chaotic traffic all contribute to a poor pedestrian walking experience. In comparison, the scattered low-value points in the area north of Snake Mountain are primarily urban areas to be renewed. These are streets surrounded by old communities and low-end businesses, with outdated infrastructure, heavy traffic, and environmental pressure. Therefore, the old town of Wuchang is not a single cluster of high-quality or low-quality streets, but two kinds of street-quality streets are evenly matched, high and low quality are mixed and distributed.

#### Measurement Results of 5D Dimensions of Physical Environment

**Figure 6:**
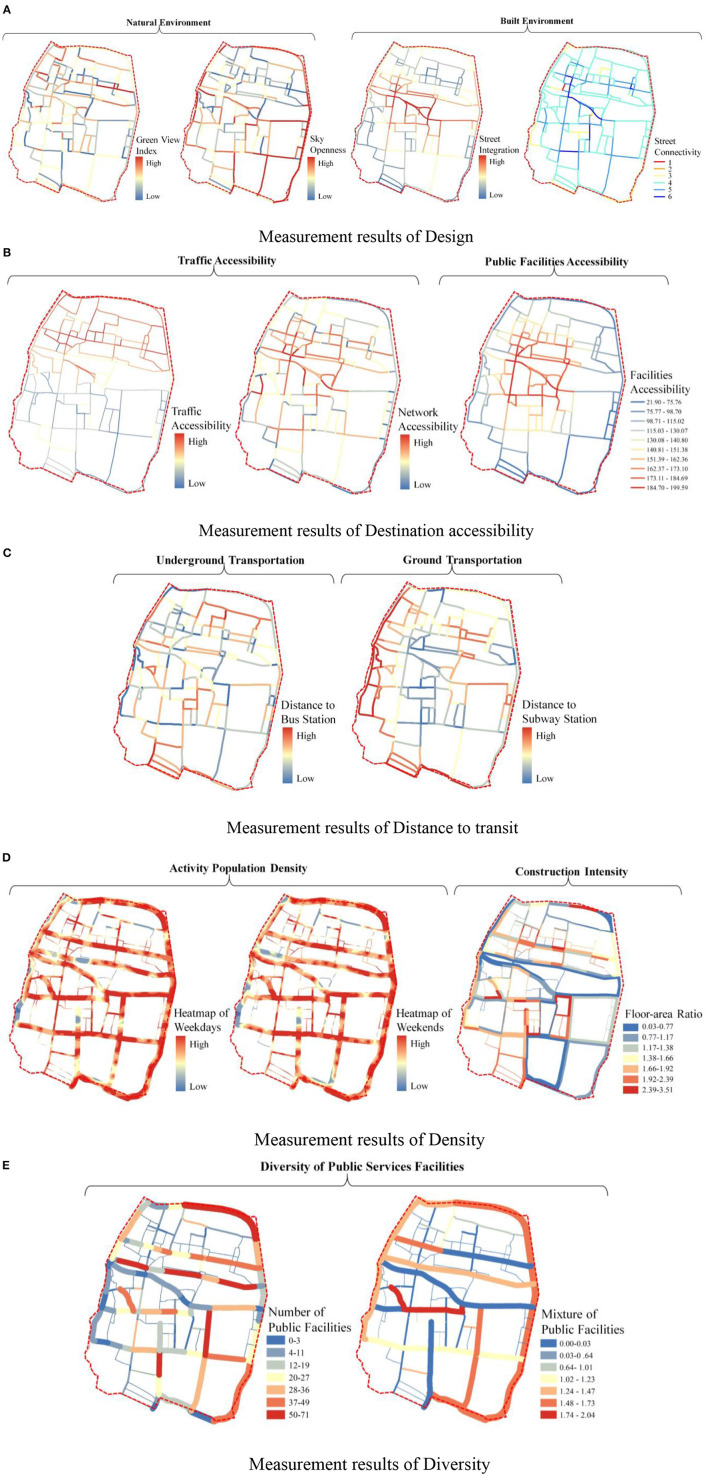
Results of the 5D dimensions physical environment quality measurement of street space. **(A)** Measurement results of design. **(B)** Measurement results of destination accessibility. **(C)** Measurement results of distance to transit. **(D)** Measurement results of density. **(E)** Measurement results of diversity.

The following results are obtained on the 5D+3S measure of the 5D dimensions in the old town:

1. In the Design measurement level, each street space's Green View Index and Sky Openness are better. The Street Integration is higher, but the Street Connectivity value is low, and the values of the south and north are distributed unevenly. The integration and connectivity values of the north of Snake Mountain are higher than the southern region (Figure 6A).
2. In the Destination accessibility measurement level, the overall accessibility of traffic facilities in the old town streets is relatively high. The Network Accessibility is much higher than the average level of the central area, and the high-value points of accessibility of public service facilities are more concentrated. However, there are some current problems in the old town, such as insufficient accessibility of traffic facilities in the streets and lanes, relatively low density of pedestrian road network, a significant difference in accessibility values of public service facilities, etc.s (Figure 6B).
3. In the level of distance to transit, the distance between the streets from the subway station and bus station is less than the 15-min pedestrian distance. The concentration of bus facilities in the north of Snake Mountain is greater than that in the south, and the overall bus accessibility in the old town is high (Figure 6C).
4. In the Density level measurement results, the density value of the active population in the old city remains above the medium level from 10 a.m. to 8 p.m. in the street space, the active crowd density is high, and the street vitality is good. At the same time, the building density in the street buffer zone in the old city is low, and the average floor area ratio is 1:48, so the pedestrian walking comfort is high (Figure 6D).
5. In the measurement results at the Diversity level, the number of public service facilities in most street spaces is insufficient, the types are single, and the Diversity of service groups is inadequate (Figure 6E).

Therefore, in the 5D dimensional measurement of the physical environment in the old town streets, the weak spatial service capacity, low traffic efficiency, and imperfect public service facilities system are the main problems.

#### Measurement Results of 3D Dimensions of Pedestrian Perception

**Figure 7:**
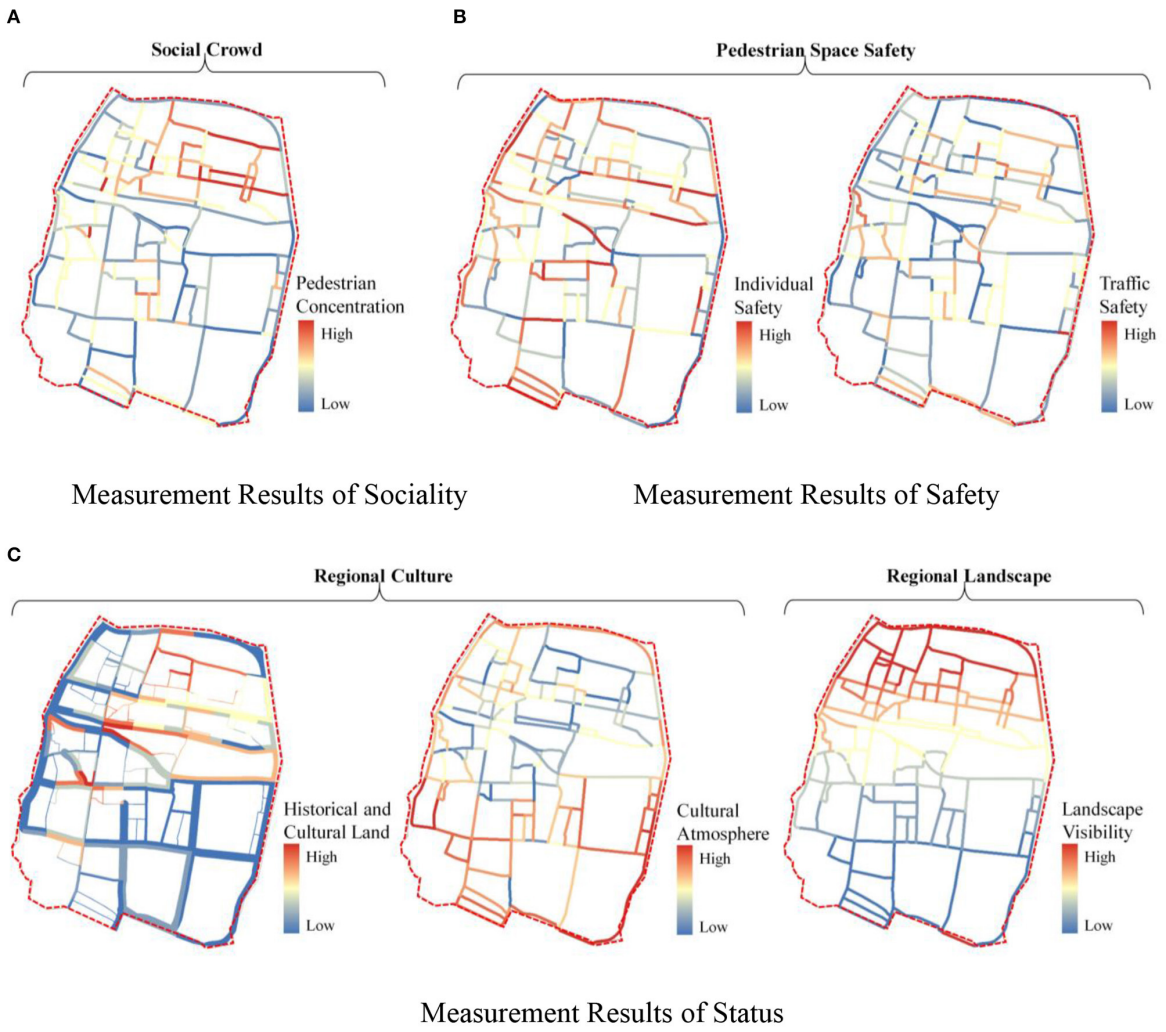
Results of the 3S dimension quality measurement of street space. **(A)** Measurement results of sociality. **(B)** Measurement results of safety. **(C)** Measurement results of status.

According to the 5D+3S quality measurement model of street space, the following results are the 3S dimensions of pedestrian perception:

1. In the results of Sociality level, the Pedestrian Concentration in the old town street space is quite good, the number of streets with medium and above concentration is up to 83%, and it is highly consistent with the distribution of historical and cultural sites, schools, and commercial space. So, the overall street vitality is good (Figure 7A).
2. In the Safety measurement results, the average value of Individual Safety is 0.048, and more than half of the streets' values are higher than the average. However, 32% of the street space in the old town is greater than the average, the high-value points are primarily distributed in the south of Snake Mountain, and pedestrian walking safety is lower (Figure 7B).
3. In the Status measurement results, the proportion of historical and cultural land in the street buffer zone, the sense of cultural atmosphere, and the value of landscape visibility decrease from north to south with Snake Mountain as the boundary, the historical and cultural land in the overall street space is relatively high. However, the values of the Cultural Atmosphere are weak, the Landscape Visibility is insufficient, and the low-value points are distributed in series. It is caused by the changes in the spatial texture of the streets and the pattern of the ancient city in the south of Snake Mountain (Figure 7C).

Therefore, the main problems are the insufficient protection of the overall street pattern of the old city and the lack of protection and display of local cultural resources.

### Analysis of Validity Test

#### Results of Objective Validity Test

**Figure 8:**
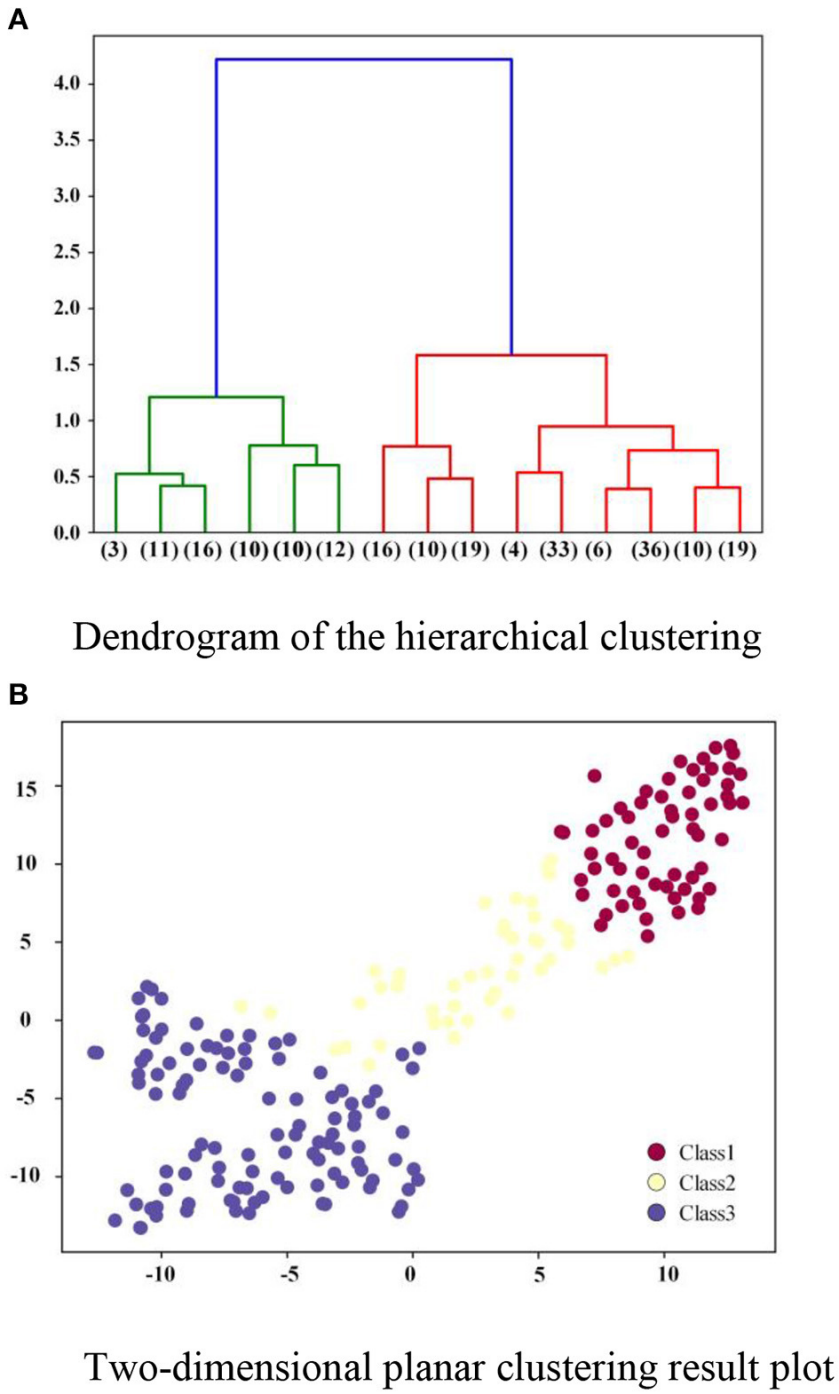
CVS clustering result graph. **(A)** Dendrogram of the hierarchical clustering. **(B)** Two-dimensional planar clustering result plot.

In the clustering process, the dendrogram of the hierarchical clustering algorithm is shown in Figure 8A, where the ordinate represents the distance between the linked clusters, and the abscissa represents the number of data points of CVS contained in the cluster represented by each node.

According to the hierarchical clustering result, Class 1 contains 62 data records, Class 2 contains 45 data records, and Class 3 contains 108 data records. In order to illustrate the clustering results more clearly, a t-SNE (90) data visualization technology is applied to reduce the dimension of the original data samples to 2D data. It can be found from Figure 8B that the projection of all the street data records on the two-dimensional plane presents three clear categories, which indicates that the distribution of these samples corresponds to the three clusters in the results of a clustering algorithm.

Then, based on the results of the hierarchical clustering algorithm, the average values of each measurement index value for the street space samples in the three categories are calculated. The radar chart depicting the average values is shown in Supplementary Figure 5A. There are significant differences and separations amongst the index values of the street samples in the three clustered categories. Additionally, Supplementary Figure 5B compares some actual street maps from the three categories of street samples. The results show that the clustering results match the human perception of street space quality.

**Figure 9:**
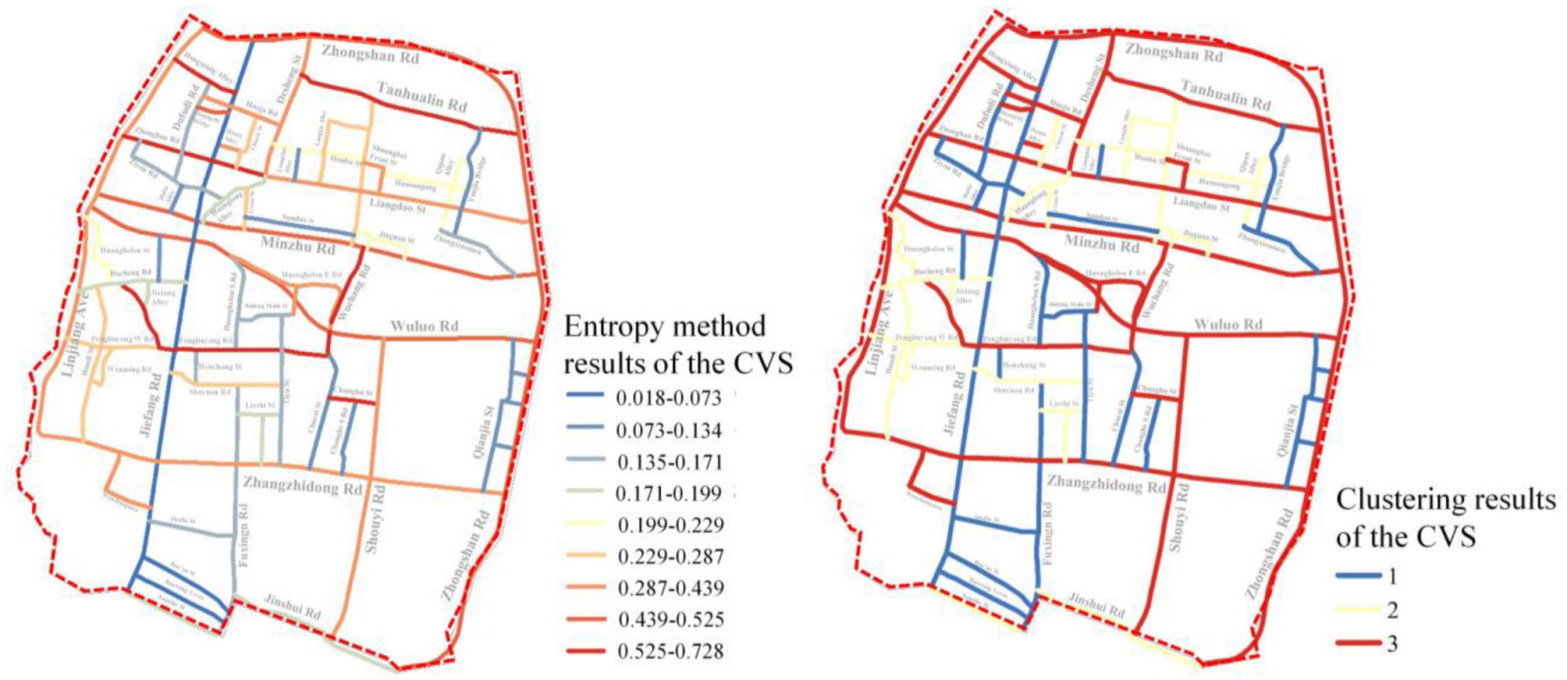
Comparison between the results of entropy method and clustering algorithm for the CVS.

Furthermore, as illustrated in Figure 9, the CVS distribution is compared to the clustering results. It can be seen that the qualitative outcome of the hierarchical clustering algorithm and the quantitative values of the measurement index obtained using the entropy weight method can also be quite similar. High-quality streets are those that have a high degree of comprehensiveness. Those with a low comprehensive value, on the other hand, are classified as low-quality.

As shown above, the proposed 5D+3S quality measurement model of street space can simultaneously grade the street quality and assign a quantitative quality score to these streets, which can be used by practitioners. The results of the preceding comparative visualization experiments also demonstrate that the proposed 5D+3S measurement model is reasonable and capable of accurately determining the street space quality.

#### Results of Subjective and Objective Comparative Validity Test

As mentioned in section Subjective and Objective Comparative Validity Test, according to the sentiment analysis and hot word extraction on the text database of public evaluation, all the streets can be divided separately with positive, negative, and neutral sentiments. The top 5 streets in popularity are selected as the studied samples, as listed in Supplementary Table 2. Among them, the street with the highest evaluation is Zhongshan Road, and the street with the lowest evaluation is Luoyu Road. However, the medium-quality streets without good or bad evaluation include Sanyang Road, Nanhu Road, and Youyi Avenue. For the sake of fairness, Youyi Road is selected randomly to represent medium-quality streets. The final three sample streets for expert evaluation are Zhongshan Road, Luoyu Road, and Youyi Road.

First, in terms of expert scoring, this study selected ten local students with a master's degree and ten practitioners in urban planning to establish an expert evaluation team. The specific scoring criteria are based on the following guidelines: Street Planning and Design Guidelines in Wuhan Elements of a Complete Streets Policy, Global Street Design Guide, and the existing highly reliable expert scoring standards (53, 91, 92). According to the scoring criteria shown in Table 3, the quality of the selected sample street is scored from physical environment and pedestrian perception.

**Table 3:** The criteria for expert scoring.

**Dimension**	**Criteria for evaluation**	**Grading scale**
Physical environment	• Street greening and sky view • Accessibility of transportation facilities • Road network accessibility • Diversity and convenience of public service facilities • Activity population density • Building density in the surrounding area.	5-Excellent (Very strong advantages with negligible weaknesses) 4-Good (Strong advantages with minor weaknesses) 3-Average (Streets have some strengths but moderate weaknesses)
Pedestrian perception	• Concentration degree of walking and riding people. • Completeness of physical warning and monitoring facilities. • Degree of motor vehicle interference. • Availability of historical and cultural facilities within a 15-minute walk. • Visibility of mountains, water bodies, and historical and cultural buildings.	2-Weak (Streets have few strengths with major weaknesses) 1-Poor (Streets have very few strengths with numerous major weaknesses)

Then, this study collects the relevant data about the three streets for objective verification, and the CVS of sample streets are calculated using the 5D+3S measurement model. The results of public evaluation, expert scoring, and CVS are comprehensively compared. It can be seen from Table 4 that the results of public evaluation, expert scoring, and 5D+3S measurement are consistent within a certain error range. The expert scoring of the street with good public evaluation is also high, and the measurement value obtained by the measurement model is also high. This consistency shows that the results of the measurement model are the same as the subjective evaluation of an individual, which proves the effectiveness of the proposed 5D+3S model.

**Table 4:** The results of the subjective evaluation and the objective validation.

**Sample street**	**Expert scoring**			**Public evaluation**	**CVS**
	**Physical environment**	**Pedestrian perception**	**Total score**		
Zhongshan road	4.1	4.5	8.6	High	0.6832
Luoyu road	1.8	1.4	3.2	Low	0.1274
Youyi road	2.8	2.7	5.5	Medium	0.2519

Therefore, the measurement model can reflect the influencing factors of subjective judgment in evaluating the quality of street space. Meanwhile, the measurement model has no human intervention in the calculation, which can exclude the impact of human subjective emotions on the quality judgment and make the measurement results more objective and convincing.

Although this study only selected three streets in the subjective verification, the research points were sampled from these three streets according to a certain distance interval. Therefore, the total number of sampling points in the verification process is relatively sufficient, which can verify the effectiveness of the measurement model to a certain extent.

Therefore, the spatial quality levels corresponding to the comprehensive values of different street space quality measures are determined according to the comparison results, and the specific corresponding relationships are shown in Table 5.

**Table 5:** Classification table of comprehensive quality measurement values of street space.

**Hierarchical clustering results**	**Measure the comprehensive value interval**	**Quality grade**
Class 1	[0.0180, 0.1714]	Low quality
Class 2	[0.1715, 0.2874]	Medium quality
Class 3	[0.2875, 0.7280]	High quality

## Discussion

### Academic Contributions

This study contributes to the study of urban space for the following reasons. First, the 5D+3S street space quality measurement model incorporates the existing 5D dimensions from the built environment measurement model (24) and the 3S dimensions were derived from the existing pedestrian perception literature. Theoretically, this model transcends the original research dimension of measuring the material space environment. It also aligns with the dual connotation of material external space and the subjective suitability evaluation associated with street space quality measurement.

Second, the open geospatial data platform collects the research data, integrating 3D spatial, 2D geographic plane, and static street view image data for street space analysis. It enriches the data types used in the street space research and serves as a reference for a large-scale street space quality research. This study analyzes data using a semantic segmentation algorithm for street view images and a hierarchical clustering algorithm for comprehensive measurement results. It is a technical guide for collecting and processing a large-scale street spatial quality data. Thus, by taking the advantage of multi-source big data and machine learning algorithms, this research presents a guideline for enhancing the precision and the integrity of street space evaluation.

Finally, as part of the model's validation, this study collects social media data on the activities, thoughts, and behaviors of individual users in a variety of locations and regions for public evaluation, overcoming the limitations of subjective evaluation methods such as expert scoring, questionnaire survey, and interview. This study uses text sentiment analysis to reveal the public's subjective evaluation of personal emotion, material environment, social culture, and other aspects of the urban environment. Due to the advantages of large data sets and high precision, it reduces the cognitive deviation in public subjective evaluations and improves the reliability of objective verification results for the 5D+3S model.

### Potential Applications in Planning and Policy

Wuhan is currently in the transitional stage of post-industrialization, between the middle and late stages. Industrial civilization is in the process of evolving into an ecological civilization. Improving the quality of public space in the city will help facilitate the urban transformation, strengthen the comprehensive management capability of the city, address short-term issues of the living environment and livelihood security, and conform to the modern requirements and trends of Wuhan. Thus, as the most heavily utilized public space of the city, the diverse street space is inextricably linked to the environmental quality and humanistic charm of the city.

The 5D+3S model of street space quality measurement is used in this study to determine the 5D dimensions of material space in 62 streets throughout the old city of Wuchang. The study concludes that the current problems of the city include inadequate street space service capacity, inefficient traffic flow, and an inefficient public service facility system. To improve the future material space quality of street space, Wuchang's old city should prioritize improving the rational traffic organization, compound street function optimization, and the construction of a diverse and perfect community life circle, in line with Wuhan's planning requirements for a 15-min community life circle.

Second, by analyzing the 3S dimension measurement results, we can identify the current issues of inadequate street pattern protection and lack of landscape, historical, and cultural resource protection and display. As a result, the top-level design of Wuchang old town should be optimized and improved in the future. The strategies can include protecting the non-renewable cultural gene pool, preserving the spatial pattern of the ancient city, and preserving the context for improving the street environment.

Simultaneously, based on the regional characteristics of the old town, there are other urban core areas that retain some traditional urban spatial patterns but face contemporary issues such as urban renewal and protection, regional vitality revival, and characteristic display. These areas are Nanjing's old city, Suzhou's ancient city, and Hefei Ring Park. As a result, the quality measurement model of street space and policy recommendations have some relevance in these areas.

### Deficiencies and Prospects

First, the 5D+3S quality measurement model of the street space proposed in this study comprehensively considers the objective and subjective factors and enriches the classical 5D model of the built environment measurement from the physical environment and pedestrian perception dimensions. However, since the quality influence factors of street space such as policy, management, and maintenance are difficult to measure and to quantify, the integrity of the measurement model and the representativeness of the index system proposed in this study need to be further optimized.

In addition, the street space of the old town in Wuchang has been selected as the research object to verify the effectiveness of the proposed measurement model. However, due to the unavoidable limitations of the old town's emergence, development, and decline, whether the measurement model can be applied to other regions and cities remains verified. Limited by the experimental conditions, this study only selected three streets to conduct the subjective verification experiments. More streets should be added in future research to further verify the model proposed in this study.

In the future, a multi-type street space in different cities will be increased. More research samples will be included to explore the street space's internal quality influence factors to build a more applicable quality measurement model of street space.

## Conclusion

With the research on the quality measurement theoretical basis of street space, this study selects the quality measurement specific indicators of street space, and constructs the 5D+3S comprehensive measurement model supported by big data technology. The old town of Wuchang is taken as the research area to measure the quality of street space in multiple dimensions with the proposed measurement model. The comparison results show that the measurement model effectively evaluates the quality of street space objectively and without human intervention. The polarization trend of the comprehensive measurement results proved the huge difference in the quality of the streets bordered by Snake Mountain in the study area. Then, the measurement indicator results show that the introduction of humanism 3S indicator also confirms the fact that insufficient street protection in the old city leads to poor pedestrian perception experience in the streets. Finally, the verification experiment results prove the effectiveness of the proposed measurement model, and the measurement results on the validation streets have a high consistency with the expert evaluation. In general, this study is a practical attempt to build a systematic quality measurement model by combining the physical environment, pedestrian vision, walking experience, and other difficult-to-measure perception indicators, which provides a new idea for the research of street space.

## Data Availability Statement

The raw data supporting the conclusions of this article will be made available by the authors, without undue reservation.

## Author Contributions

TW: contributed to conceptualization, methodology, software, investigation, resources, and data curation and was responsible for the original draft and supervision. TW and PS: were responsible for review and editing. WL and PS: contributed to visualization. All authors contributed to the manuscript revision, read, and approved the submitted version. All authors contributed to the article and approved the submitted version.

## Funding

This study was supported by the Fundamental Research Funds for the Central Universities with funding number: DUT20RC(3)051.

## Conflict of Interest

The authors declare that the research was conducted in the absence of any commercial or financial relationships that could be construed as a potential conflict of interest.

## Publisher's Note

All claims expressed in this article are solely those of the authors and do not necessarily represent those of their affiliated organizations, or those of the publisher, the editors and the reviewers. Any product that may be evaluated in this article, or claim that may be made by its manufacturer, is not guaranteed or endorsed by the publisher.
