# Supplementary material for: Constructing the Quality Measurement Model of Street Space and Its Application in the Old Town in Wuhan
Source: Front Public Health. 2022 Feb 24;10:816317. doi: 10.3389/fpubh.2022.816317 (PMC8907578; doi:10.3389/fpubh.2022.816317)
Supplement: Supplementary file 1 [file Data_Sheet_1.docx]

Supplementary Material

# Supplementary Figures


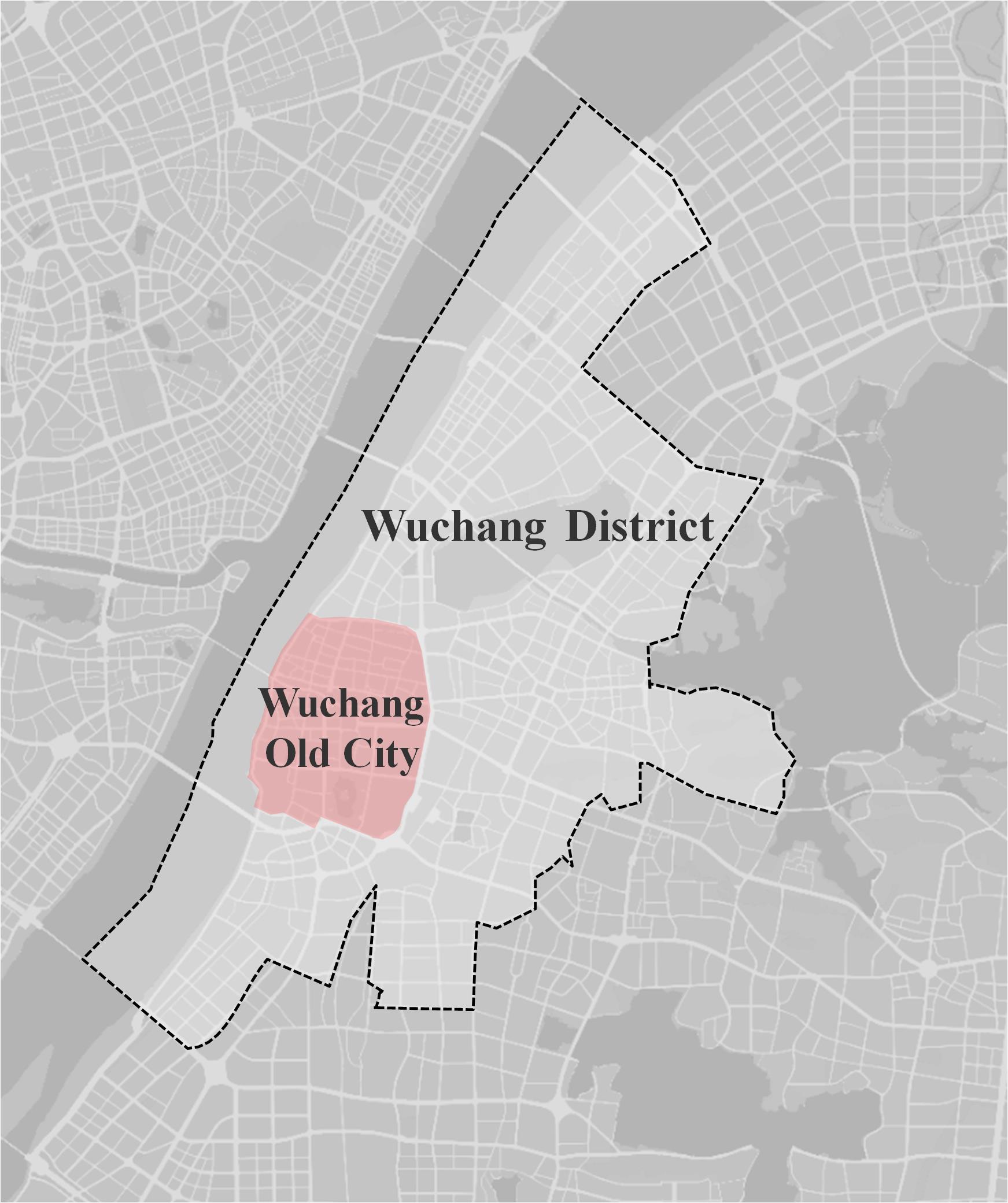


Supplementary Figure 1. Old town ground map in Wuchang City


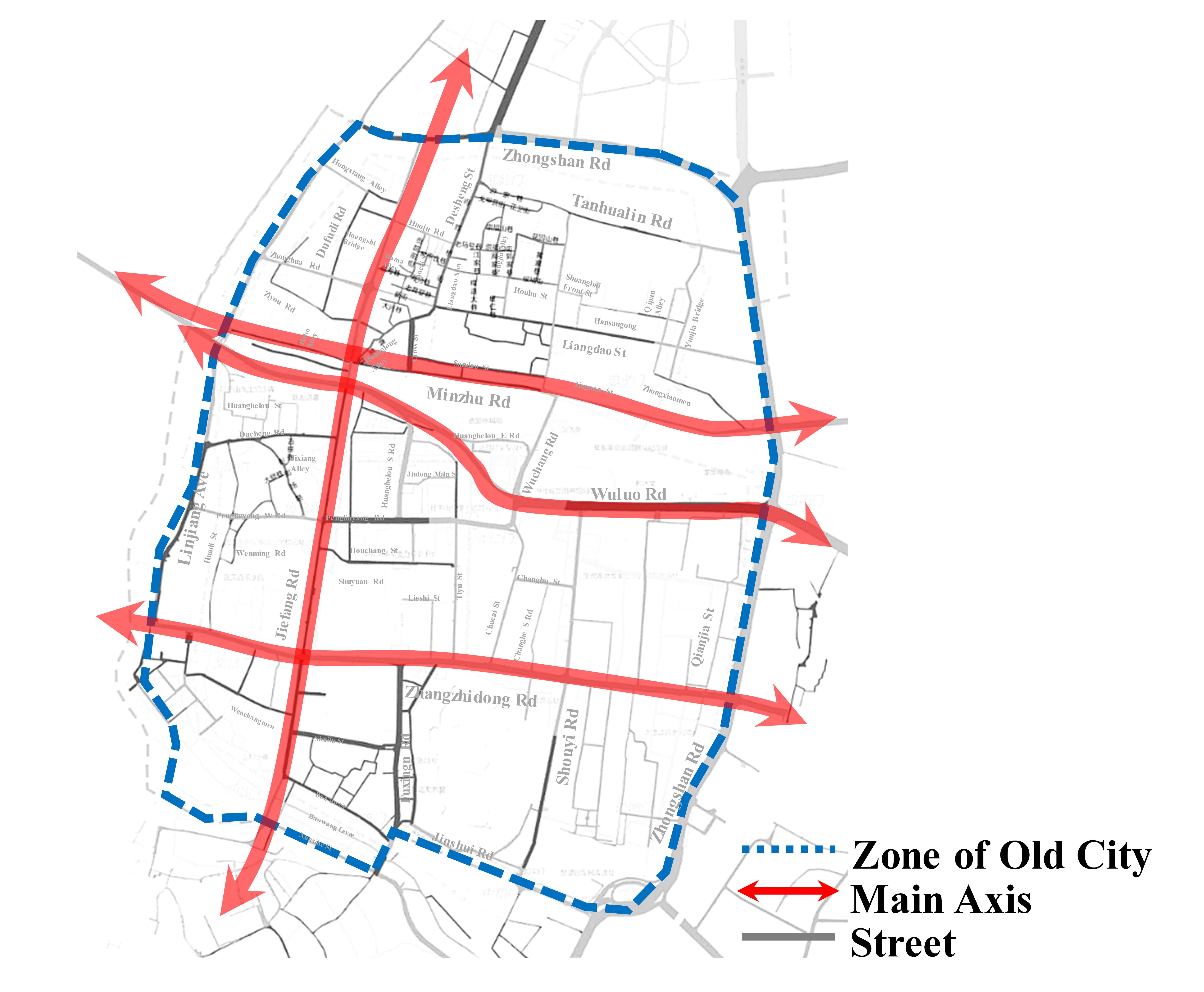


**Supplementary Figure 2.** Old town road network pattern


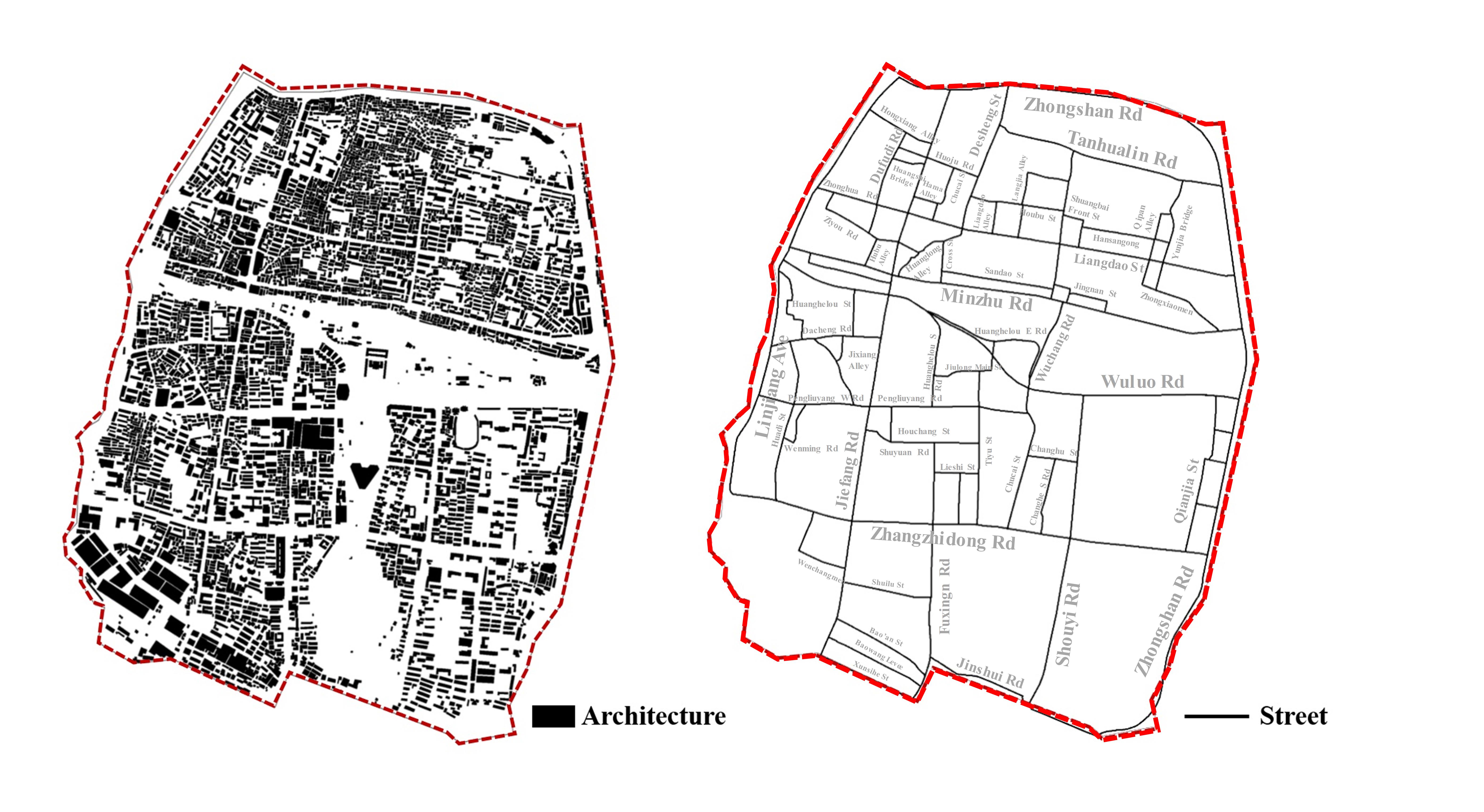


**Supplementary Figure 3.** Building data and road network data in the old town of Wuchang


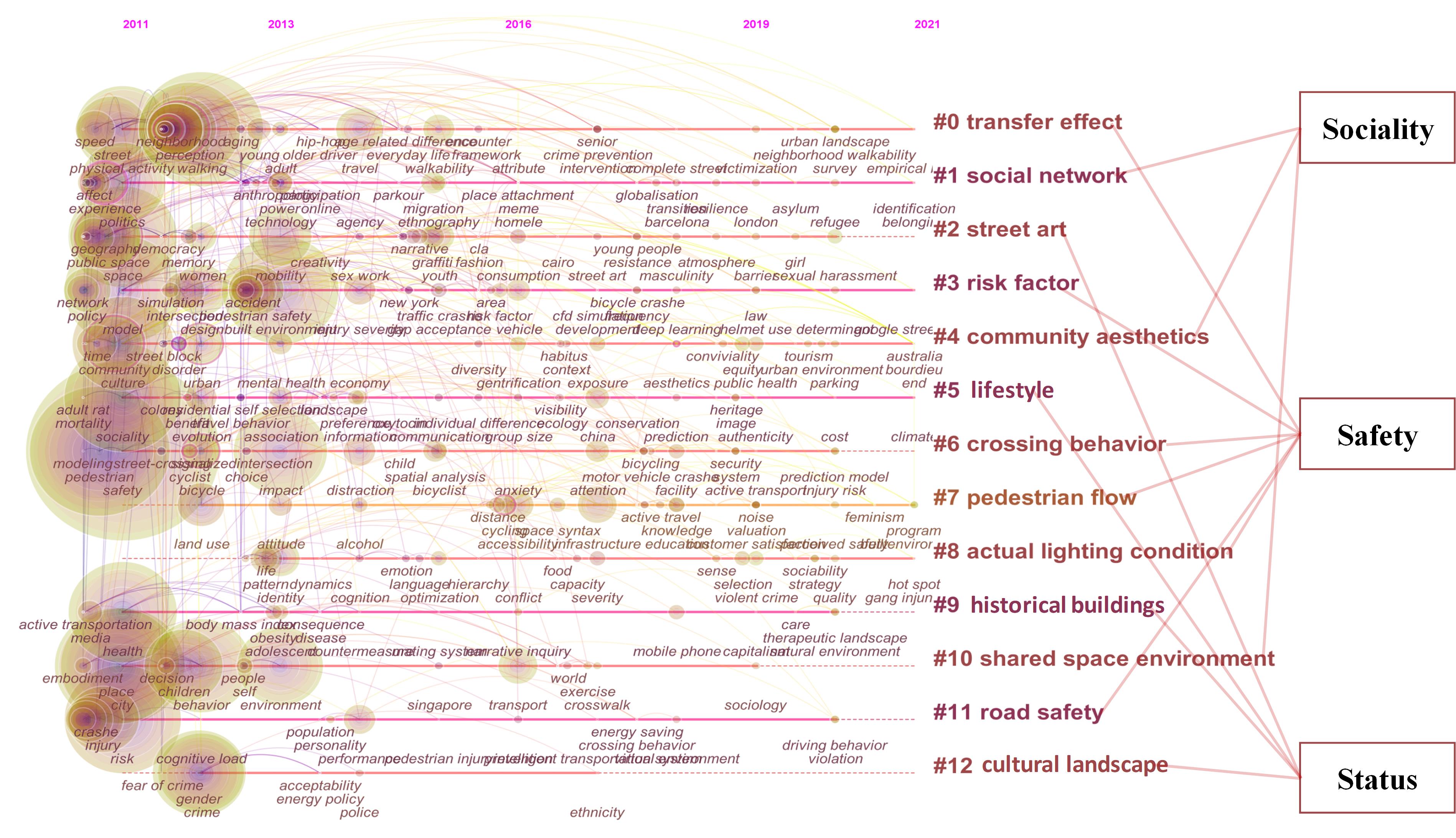


**Supplementary Figure 4.** Cluster content of subjective perception measures of street space literature


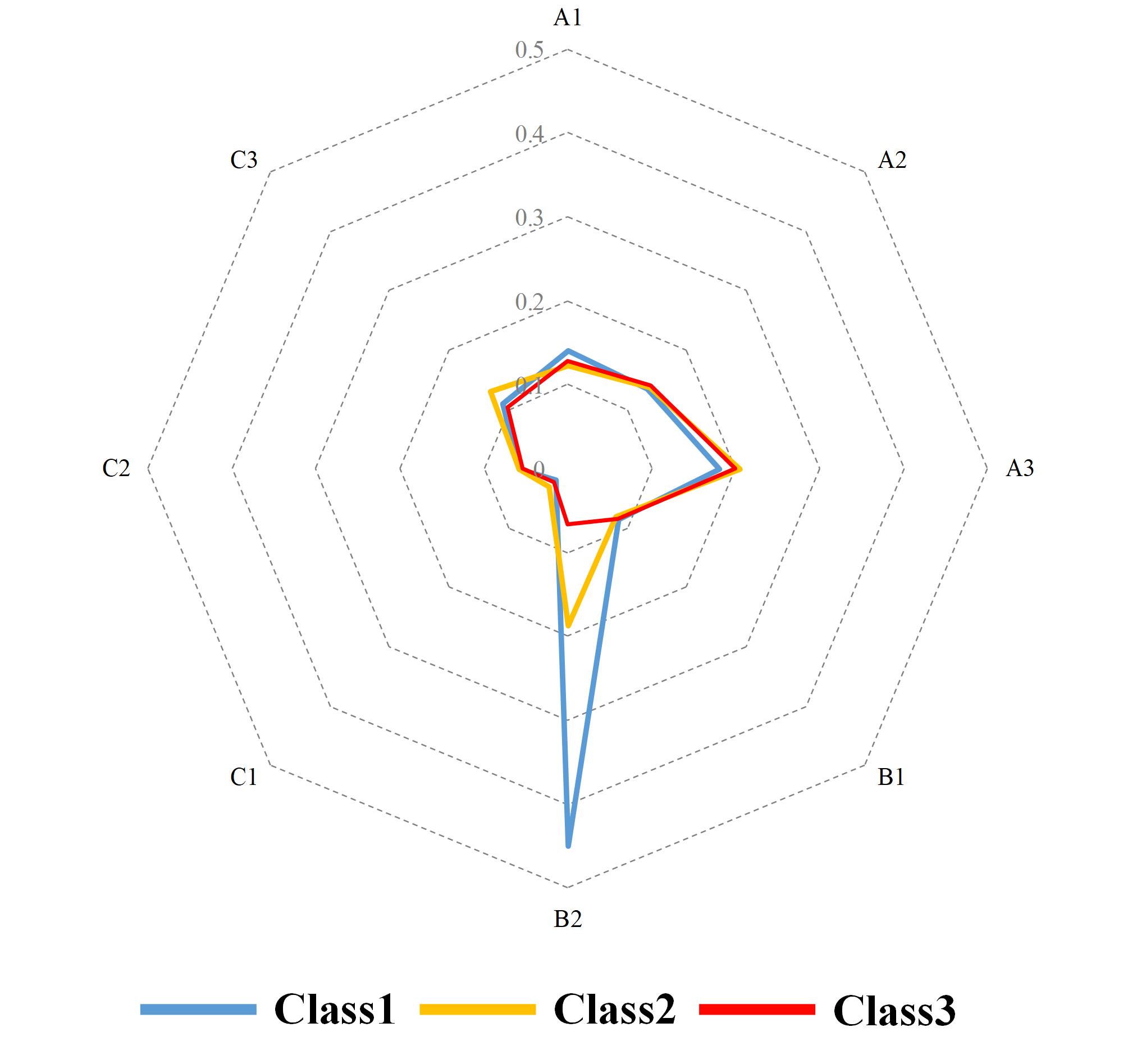


(a) Radar plot of the clustering outcome indicators


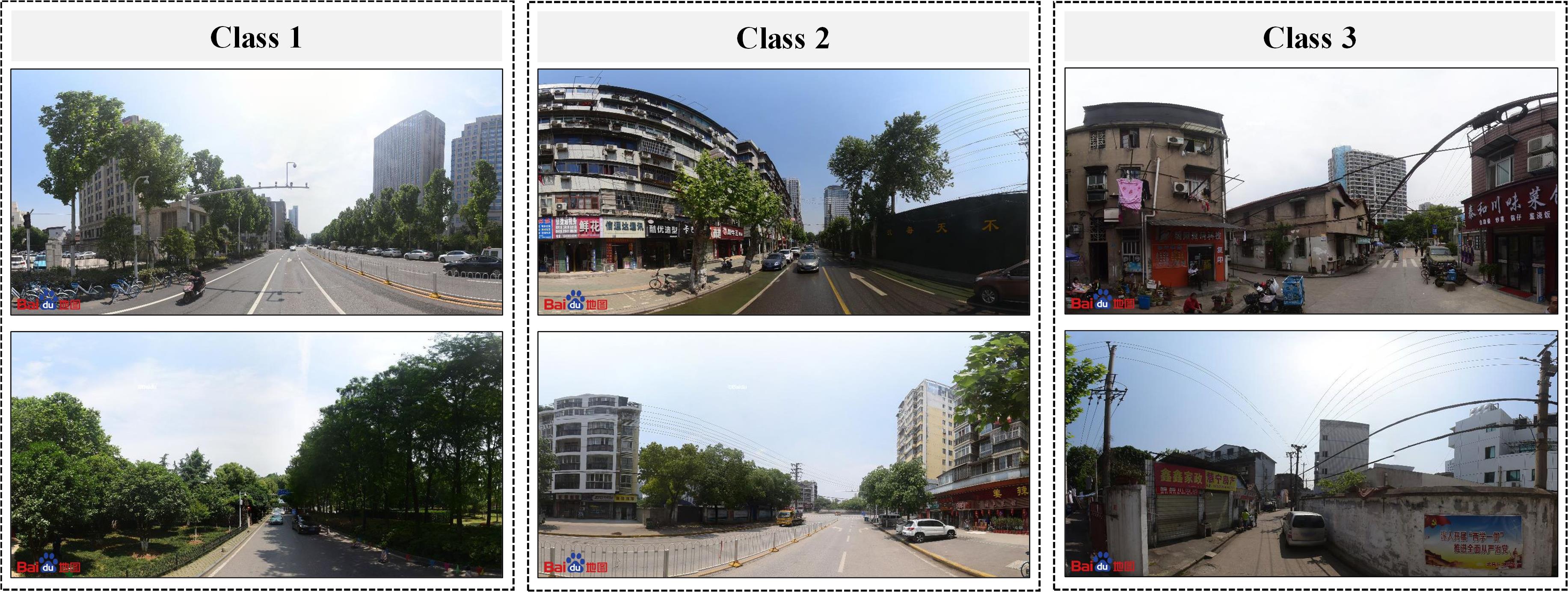


(b) Example of actual street scenes of different categories

**Supplementary Figure 5.** Comparison diagram of the clustering results and the street space real scenes

# Supplementary Tables

**Supplementary Table 1.** Street sampling points for different types of neighborhoods

| **Neighborhood type** | Little neighborhood | Middle neighborhood | Superblock |
| --- | --- | --- | --- |
| **Neighborhood scale** | 1-16 hectare | 16-36 hectare | More than 36 hectares |
| **Sampling spacing** | 150 m | 300 m | 500 m |
| **Take point distribution** | 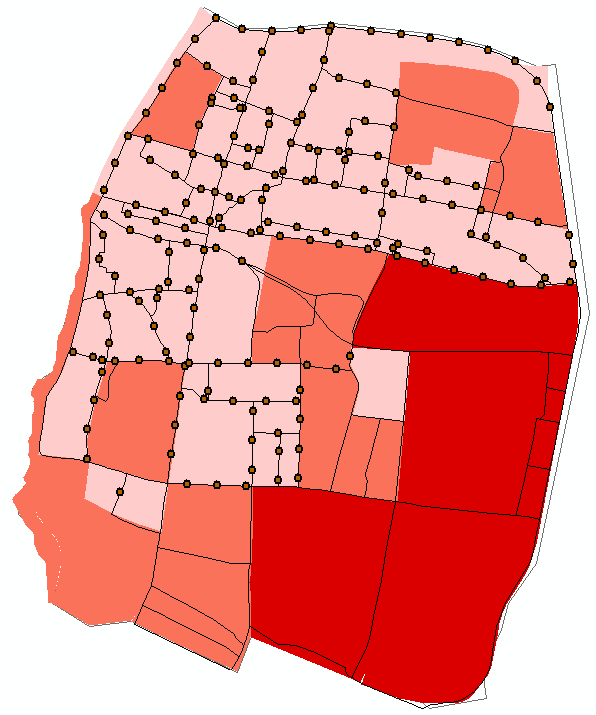 | 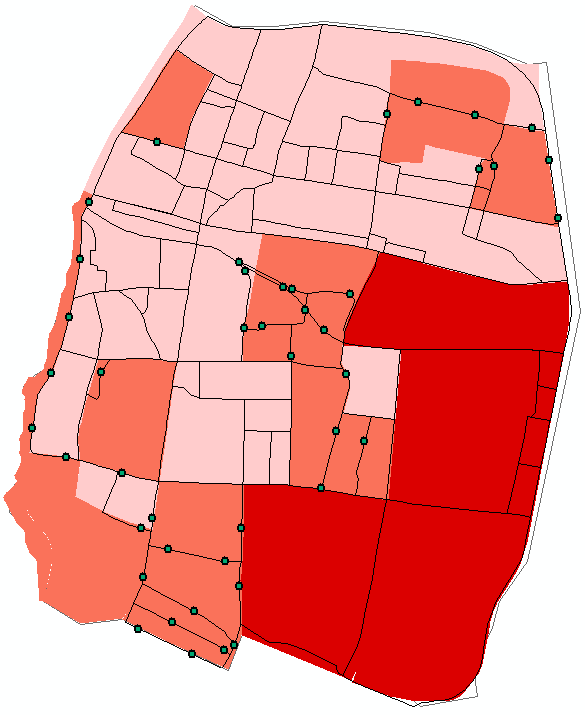 | 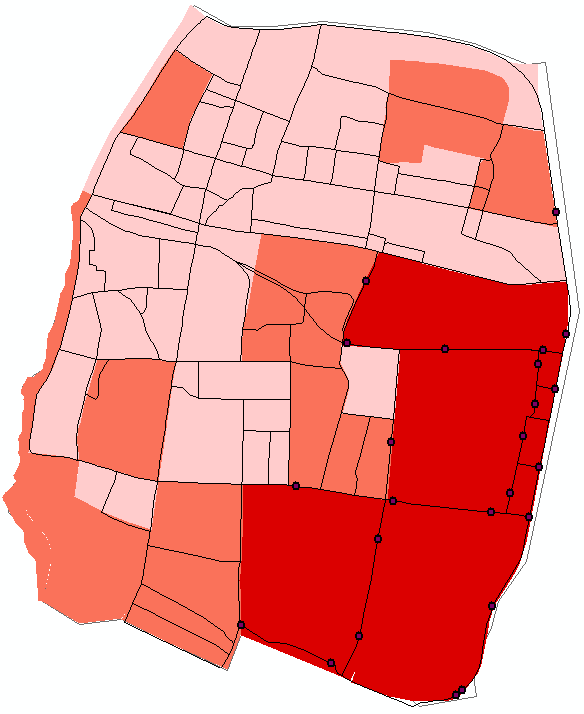 |

**Supplementary Table 2.** Results of public evaluation of street space quality

| **Ranking** | **Positive Street** | **Negative Street** | **Neutral Street** |
| --- | --- | --- | --- |
| 1 | Zhongshan Road | Luoyu Road | Sanyang Road |
| 2 | Jianghan Road | Xinhua Road | Nanhu Road |
| 3 | Lihuangpi Road | Dongguan Road | Youyi Avenue |
| 4 | Yanjiang Avenue | Zhangjiawan Street | Heping Road |
| 5 | Dazhi Road | Wulidun Road | Xudong Avenue |
